# Massive Autophosphorylation of the Ser/Thr-Rich Domain Controls Protein Kinase Activity of TRPM6 and TRPM7

**DOI:** 10.1371/journal.pone.0001876

**Published:** 2008-03-26

**Authors:** Kristopher Clark, Jeroen Middelbeek, Nick A. Morrice, Carl G. Figdor, Edwin Lasonder, Frank N. van Leeuwen

**Affiliations:** 1 Department of Tumor Immunology, Nijmegen Centre for Molecular Life Sciences, Radboud University Nijmegen Medical Centre, Nijmegen, The Netherlands; 2 Laboratory of Pediatric Oncology, Nijmegen Centre for Molecular Life Sciences, Radboud University Nijmegen Medical Centre, Nijmegen, The Netherlands; 3 Centre for Molecular and Biomolecular Informatics, Nijmegen Centre for Molecular Life Sciences, Radboud University Nijmegen Medical Centre, Nijmegen, The Netherlands; 4 University of Dundee, MRC Protein Phosphorylation Unit, James Black Centre, Dundee, Scotland, United Kingdom; University of Oldenburg, Germany

## Abstract

TRPM6 and TRPM7 are bifunctional proteins expressing a TRP channel fused to an atypical α-kinase domain. While the gating properties of TRPM6 and TRPM7 channels have been studied in detail, little is known about the mechanisms regulating kinase activity. Recently, we found that TRPM7 associates with its substrate myosin II via a kinase-dependent mechanism suggesting a role for autophosphorylation in substrate recognition. Here, we demonstrate that the cytosolic C-terminus of TRPM7 undergoes massive autophosphorylation (32±4 mol/mol), which strongly increases the rate of substrate phosphorylation. Phosphomapping by mass spectrometry indicates that the majority of autophosphorylation sites (37 out of 46) map to a Ser/Thr-rich region immediately N-terminal of the catalytic domain. Deletion of this region prevents substrate phosphorylation without affecting intrinsic catalytic activity suggesting that the Ser/Thr-rich domain contributes to substrate recognition. Surprisingly, the TRPM6-kinase is regulated by an analogous mechanism despite a lack of sequence conservation with the TRPM7 Ser/Thr-rich domain. In conclusion, our findings support a model where massive autophosphorylation outside the catalytic domain of TRPM6 and TRPM7 may facilitate kinase-substrate interactions leading to enhanced phosphorylation of those substrates.

## Introduction

The channel-kinases TRPM6 and TRPM7 are bifunctional proteins consisting of a TRP cation channel fused to a kinase and represent the only two proteins in the mammalian genome with this particular architecture [1], [2]. A critical issue in understanding these intriguing proteins is to identify the molecular mechanisms controlling the activation of the channel as well as kinase activity and establish the functional link between these two domains.

Within only a few years, major advances have been made in our understanding of the role and regulation of TRPM6 and TRPM7 channels. Genetic and electrophysiological studies have identified a major role for TRPM6 and TRPM7 in Mg^2+^ homeostasis. Both TRPM6 and TRPM7 channels permeate divalent cations including Mg^2+^ [3]–[5]. Mutations in TRPM6 cause the disease familial hypomagnesemia with secondary hypocalcemia [6], [7] whereas ablation of TRPM7 in DT-40B cells leads to growth arrest as the cells become hypomagnesic [8]. Importantly, these phenotypes can be rescued by supplementing the patient's diet or growth media with Mg^2+^ [6]–[8]. However, recent evidence suggests additional cellular roles for TRPM6 and TRPM7. For instance, TRPM7 has been implicated in various cellular functions, many of which are dependent on Ca^2+^-influx, including cell proliferation, anoxic cell death, exocytosis, mechanotransduction, actomyosin contractility and cell adhesion [3], [8]–[15].

The principal feature of TRPM6 and TRPM7 channels, which distinguishes them from other TRP channels, is their sensitivity to physiological Mg^2+^ and Mg^2+^-nucleotide concentrations [3], [5], [16]. These channels are activated upon depletion of Mg^2+^ ions in cells and native TRPM7-like currents have been called magnesium inhibited cation (MIC) or magnesium-nucleotide regulated metal ion (MagNuM) currents [3], [17]. This property suggested a role for the kinase in regulating the gating of these channels. However, it has become evident that catalytic activity is not required for TRPM7 channel opening although the kinase domain may govern the set point for Mg^2+^ sensitivity [8], [18], [19]. We and others have found that TRPM7 channels are also regulated by receptor-mediated signaling [20]–[22]. While the function and regulation of TRPM6 and TRPM7 channels have been extensively studied, little is known about the molecular mechanisms activating their kinase moieties.

TRPM6 and TRPM7 are members of a novel family of atypical protein kinases called α-kinases [23], [24]. These kinases display little sequence similarity with conventional protein kinases (CPK). Moreover, α-kinases can be further distinguished from CPK by their substrate specificity. While CPK phosphorylate amino acids found in irregular structures as well as turns and loops [25], α-kinases are thought to predominantly phosphorylate residues present in α-helices [26]. Despite these differences, the topology of the α-kinase domain is very similar to that of CPK [27].

Like CPK, TRPM6 and TRPM7 undergo autophosphorylation [18], [28], [29] but the functional consequences for catalytic activity are unknown. Recently, we demonstrated that the interaction between TRPM7 and the actomyosin cytoskeleton is dependent on kinase activity, suggesting a role for autophosphorylation in the recognition of its substrates including myosin II [10]. Therefore, we further investigated the role of autophosphorylation in regulating the activity of the mammalian α-kinases TRPM6 and TRPM7.

## Results

### TRPM7 undergoes massive autophosphorylation of its C-terminus

**Figure 1:**
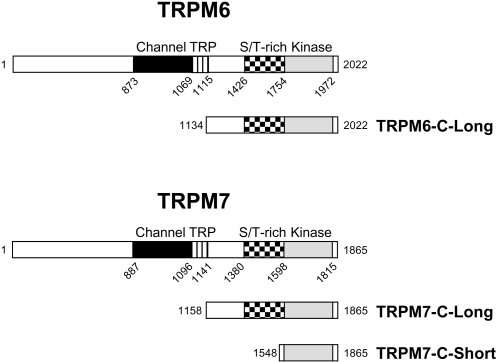
Schematic diagram depicting the constructs of TRPM6 and TRPM7 used in the current investigation. TRPM6 and TRPM7 encode both a channel (black) and an α-kinase domain (grey). The typical TRP domain (stripes) is present directly C-terminal to the last transmembrane domain and a Ser/Thr-rich region (checkered) is present immediately N-terminal of the kinase domain. In addition to the full-length protein, the entire cytosolic C tail but excluding the TRP domain of TRPM6 and TRPM7 (TRPM6-C-long and TRPM7-C-long) as well as the isolated kinase domain of TRPM7 (TRPM7-C-short) were expressed as HA-tagged recombinant proteins in mammalian cells. Mouse and human TRPM7 sequences found in the NCBI database differ by a single amino acid. Since we used human TRPM7 in this study, we have corrected all positions to correspond to the human sequence and therefore Ser1511 and Ser1567 become Ser1512 and Ser1568, respectively.

**Figure 2:**
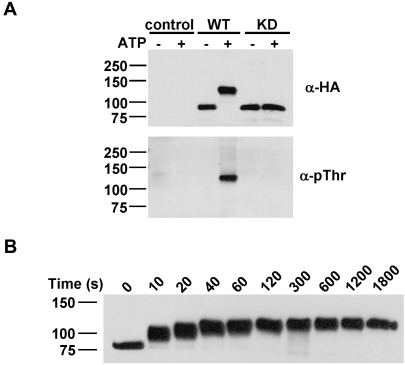
TRPM7 undergoes extensive autophosphorylation of the C-terminus via a sequential series of phosphorylation events. (A) Autophosphorylation of TRPM7 dramatically reduces its electrophoretic mobility. WT and KD TRPM7-C-long were incubated in the absence or presence of MnATP for 30 min and detected using anti-HA (top panel) and anti-pThr antibodies (bottom panel). (B) Kinetics of TRPM7 autophosphorylation. TRPM7-C-long was incubated with MnATP for the indicated times. Proteins were detected by immunoblotting using anti-HA antibodies.

To test the role of autophosphorylation in modulating the kinase activity of TRPM7, the cytosolic C-terminal tail of TRPM7 (TRPM7-C-long; aa 1158–1864; Fig. 1) was purified from mammalian cells and subjected to *in vitro* kinase assays. Remarkably, incubation of TRPM7 with ATP *in vitro* led to the incorporation of 32±4 mol of phosphate per mol of kinase which was accompanied by a dramatic shift in electrophoretic mobility on SDS-PAGE gels (Fig. 2A). The non-phosphorylated protein, which migrates at around 80 kDa, shows an apparent molecular mass of 120 kDa after *in vitro* kinase assays. The change in electrophoretic mobility is due to autophosphorylation because incubation of a catalytically inactive mutant with ATP had no effect on the migratory behavior of TRPM7-C-long. The kinetics of TRPM7-C-long autophosphorylation demonstrates that this process is very rapid. Autophosphorylation was detectable within 10 s and reached completion within 2-5 min (Fig. 2B). Moreover, autophosphorylation of TRPM7-C-long proceeds via multiple intermediate phosphorylation states as depicted by the gradual change in electrophoretic mobility of TRPM7. These results demonstrate that TRPM7 massively phosphorylates its cytosolic C-terminal tail via a multi-step process.

### The change in electrophoretic mobility of TRPM7 correlates with its ability to phosphorylate exogenous substrates

**Figure 3:**
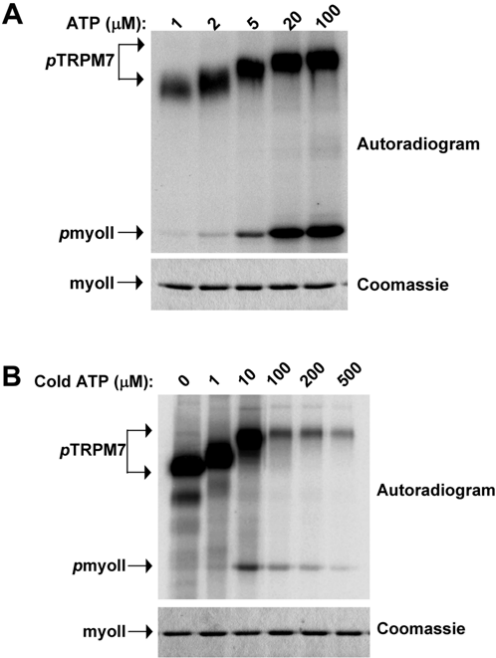
Onset of substrate phosphorylation occurs after completion of TRPM7 autophosphorylation. (A) Effect of reducing ATP concentration on TRPM7 autophosphorylation and myosin II phosphorylation. TRPM7-C-long was incubated with GST-myosin IIA in the presence of varying MnATP concentrations for 30 min at 30°C. The specific activity of ATP was maintained constant for all samples. The proteins were separated by SDS-PAGE, stained with coomassie brilliant blue (bottom panel) and phosphorylated proteins were detected by autoradiography (top panel). (B) Myosin II phosphorylation correlates with TRPM7 autophosphorylation even at exceedingly high specific activities. The experiment was performed as described in A except that the specific activity of the ATP solution was varied to detect myosin II phosphorylation at low ATP concentrations. All samples contained 5 µCi of [γ−^32^P]-ATP supplemented with the indicated concentrations of cold ATP.

**Figure 4:**
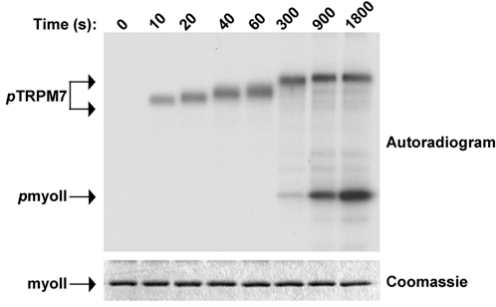
TRPM7 autophosphorylation precedes phosphorylation of substrates. TRPM7-C-long was incubated with GST-myosin IIB in the presence of Mn[γ−^32^P]-ATP for the indicated times. The proteins were separated by SDS-PAGE, revealed by coommassie staining (bottom panel) and phosphorylated proteins were detected by autoradiography (top panel).

The degree of TRPM7 autophosphorylation depends on ATP concentration (Fig. 3). Titration of ATP demonstrated that TRPM7-C-long undergoes complete autophosphorylation at concentrations ranging between 10 and 500 µM when using Mn^2+^ as cofactor. Notably, the phosphorylation of myosin II was optimal at those ATP concentrations that led to complete autophosphorylation of TRPM7-C-long (Fig. 3A). Even at very high [γ-^32^P]ATP specific activities, phosphorylation of myosin II was not observed when ATP concentrations were below a certain threshold (10 µM) (Fig. 3B). In contrast, TRPM7-C-long autophosphorylation was easily detected at these low ATP concentrations (Fig. 3B). When comparing the kinetics of kinase versus substrate phosphorylation, we observed that the onset of myosin II phosphorylation occurs when TRPM7 autophosphorylation is complete (Fig. 4). Again, TRPM7-C-long autophosphorylation reached maximum after about 5 min. In contrast, myosin II phosphorylation was only measurable after 5 min and increased throughout the experiment, which was stopped at the 30 min mark. These results indicate that TRPM7 autophosphorylation facilitates the subsequent phosphorylation of exogenous substrates.

### TRPM7 autophosphorylation accentuates the kinetics of substrate phosphorylation

**Figure 5:**
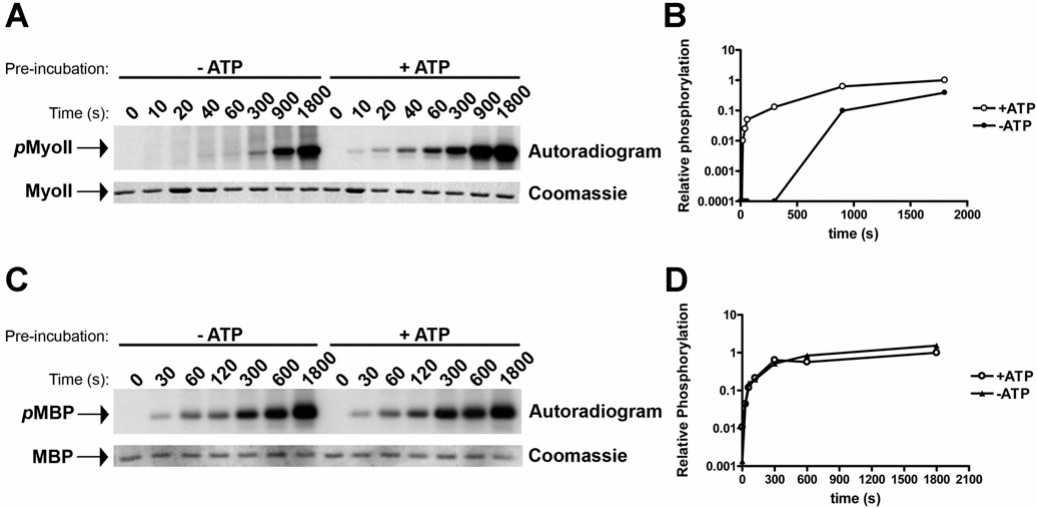
Autophosphorylation of TRPM7 accelerates susbtrate phosphorylation without affecting catalytic activity. (A) Kinetics of myosin II phosphorylation by TRPM7 prior to and after autophosphorylation. TRPM7-C-long was incubated in the absence (−ATP) or presence (+ATP) of cold MnATP to generate non-phosphorylated and completely autophosphorylated TRPM7. ATP was subsequently removed by washing the kinase with *in vitro* kinase buffer lacking ATP. Kinase reactions were initiated by adding Mn[γ−^32^P]-ATP and GST-myosin IIB and allowed to proceed at 30°C for the indicated times. Proteins were resolved by SDS-PAGE and visualized by coomassie staining (bottom panel). Phosphorylated myosin II (top panel) was detected by autoradiography. Note that the kinase pre-incubated in buffer without ATP (−ATP) must first complete autophosphorylation (5 min), apparent from the reduced electrophoretic mobility shift of TRPM7 (refer to Fig. 4), to acquire the ability to phosphorylate its substrates. (B) Quantification of myosin II phosphorylation by TRPM7 pre-incubated in absence (−ATP) or presence (+ATP) of ATP. ^32^P incorporation was measured by phosphorimaging analysis. The phosphorylation levels measured for myosin incubated for 30 min with TRPM7 that was pre-incubated with ATP was set to 1 and all other values are reported relative to that sample. (C) TRPM7 autophosphorylation does not affect catalytic activity. Experiment was performed as described in part A but using MBP as substrate. (D) Quantification of MBP phosphorylation by TRPM7 pre-incubated in absence (−ATP) or presence (+ATP) of ATP. Data was derived as described in part B.

To show that autophosphorylation regulates TRPM7 kinase activity towards its substrates, we compared the rate of myosin II phosphorylation by TRPM7-C-long before and after treatment with ATP (Fig. 5). In this classical experimental design, it is predicted that the phosphorylated kinase will phosphorylate its substrates more rapidly than the nonphosphorylated kinase if autophosphorylation is a key event in activating the protein kinase. The reason for the difference in kinetics is that the kinase pre-treated with ATP will be able to directly phosphorylate its substrates whereas the nonphosphorylated kinase will first need to undergo autophosphorylation. Here, we show that pre-treatment of TRPM7-C-long with ATP led to a significant increase in the kinetics of myosin II phosphorylation (Fig. 5A,B). Suddenly, phosphorylation of myosin II was detectable within 10 s and increased steadily in the first 5 min, which is a time scale where no to little myosin II phosphorylation was detected if TRPM7-C-long was not previously incubated with ATP. TRPM7 requires approximately 5 min to complete autophosphorylation (Fig. 4) and after this time point, myosin II phosphorylation will proceed at the same rate in both samples explaining the detection of significant myosin II phosphorylation at later time points. In contrast, phosphorylation-kinetics of the artificial substrate MBP were not affected by prior treatment of TRPM7 with ATP as previously described [29] (Fig. 5C,D). These results demonstrate that TRPM7 autophosphorylation facilitates kinase activity towards exogeneous substrates such as myosin II without affecting intrinsic catalytic activity.

### Massive autophosphorylation of the Ser/Thr-rich domain (aa 1380–1548) controls substrate phosphorylation

**Figure 6:**
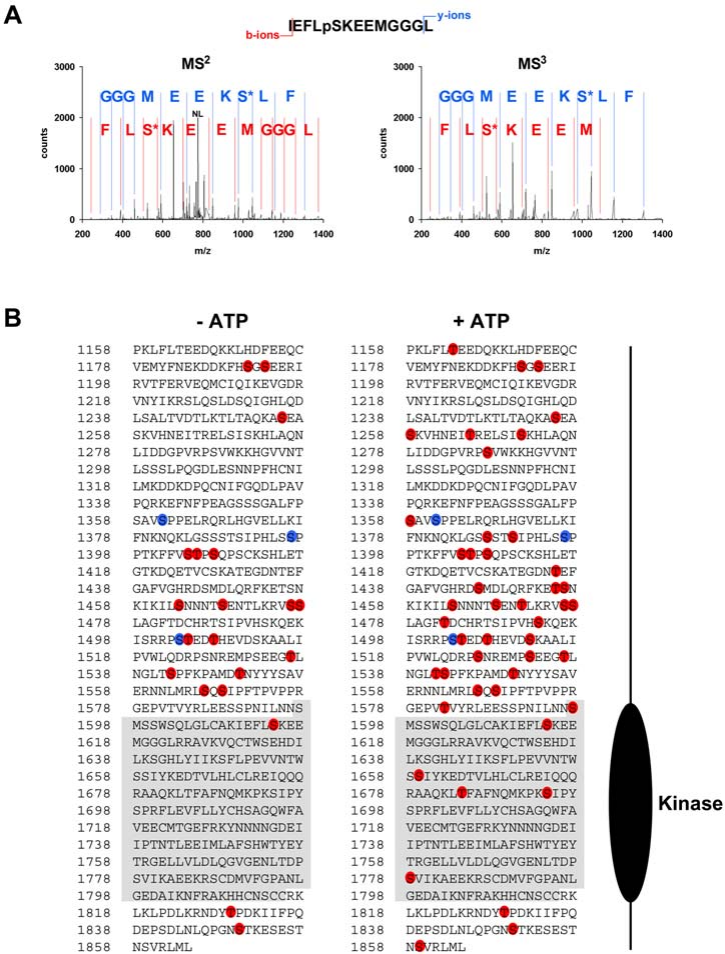
Identification of phosphorylated residues in TRPM7 by LC-MS/MS. WT and KD TRPM7-C-long were incubated in the presence of MnATP for 0 or 30 min. Tryptic peptides were analyzed using LTQ-FT mass spectrometer. (A) Representative MS^2^ and MS^3^ spectra for a TRPM7 peptide phosphorylated on Ser-1614. (m/z observed of parent ion was 823.3848, mass accuracy 1.85 ppm, +2 charge state; NL indicates neutral loss of H_3_PO_4_ which triggers acquisition of MS^3^ spectrum). The b^+^-ion series is indicated in red whereas the y^+^-ion series is in blue. S* refers to dehydroserine. (B) Diagram depicting the phosphorylated residues identified in WT (red) and KD (blue) TRPM7-C-long incubated in presence (right) or absence (left) of ATP.

To address the mechanism by which autophosphorylation affects TRPM7 kinase activity, we first mapped the phosphorylation sites by nano liquid chromatography tandem mass spectrometry (LC-MS/MS) using a LTQ-FT mass spectrometer according to a recent algorithm for post-translational modification scoring from Olsen and colleagues [30]. To discriminate between the residues phosphorylated by exogenous kinases and TRPM7 itself, a catalytically inactive mutant of TRPM7-C-long was also analyzed in these experiments. Our data identify 49 phosphorylation sites of which 3 were also found in the kinase-dead (KD) mutant (Fig. 6 and Suppl. Table S1). Thus we have identified 46 TRPM7 autophosphorylation sites and 3 sites targeted by other protein kinases. Moreover, many of the novel TRPM7 autophosphorylation sites are phosphorylated *in vivo* demonstrating the physiological relevance of the identified sites (Fig. 6B). It should be noted however that these sites are probably substoichiometrically phosphorylated *in vivo* and further phosphorylated when TRPM7 is incubated with ATP *in vitro*. Although autophosphorylation sites are present throughout the entire C-terminal tail, the majority of these sites are concentrated in the Ser/Thr-rich region directly adjacent to the catalytic domain (1380–1585). We therefore hypothesized that this region could be an important regulatory domain.

**Figure 7:**
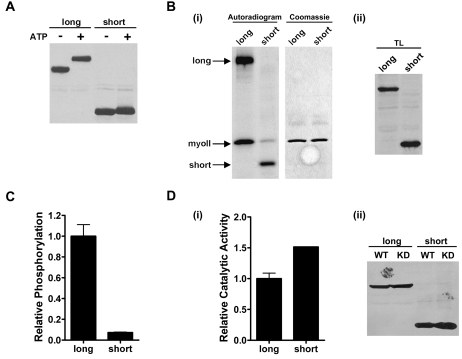
Deletion of the Ser/Thr-rich domain in TRPM7 reduces substrate phosphorylation. (A) Autophosphorylation of TRPM7-C-short only leads to a minor change in electrophoretic mobility on SDS-PAGE gels. TRPM7-C-short and TRPM7-C-long were incubated in the absence or presence of MnATP for 30 min at 30°C and subjected to immunoblotting using anti-HA antibodies. (B) TRPM7-C-short phosphorylates myosin II less efficiently than TRPM7-C-long. (i) TRPM7-C-short and TRPM7-C-long were incubated with GST-myosin IIB in the presence of Mn[γ−^32^P]-ATP. The proteins were separated by SDS-PAGE, revealed by coommassie staining (middle panel) and phosphorylated proteins were detected by autoradiography (left panel). (ii) Equal amounts of each kinase was verified by Western blotting (right panel). (C) Quantification of myosin II phosphorylation by TRPM7-C-short and TRPM7-C-long. ^32^P incorporation was measured by phosphorimaging analysis. The phosphorylation levels measured for myosin II incubated with TRPM7-C-long was set to 1 and values obtained for TRPM7-C-short are reported relative to that value. (D) Deletion of residues 1158-1548 does not affect catalytic activity. (i) TRPM7-C-long and TRPM7-C-short WT and KD were incubated with MH1 peptide in presence of Mn[γ−^32^P]-ATP. The level of phosphorylation was measured by scintillation counting. Background activity as determined using the KD mutant was substracted from the WT TRPM7 samples and catalytic activity was reported relative to that of TRPM7-C-long, which was set to 1. (ii) Equal amounts of each kinase were verified by Western blotting.

We investigated the contribution of this Ser/Thr-rich domain to TRPM7 kinase activity by generating a construct (TRPM7-C-short) containing only the catalytic domain plus a small extension (40 aa) that is required for dimerization. TRPM7-C-short autophosphorylated itself leading to the incorporation of approximately 4 times less phosphate than TRPM7-C-long. However in contrast to TRPM7-C-long, the electrophoretic mobility of TRPM7-C-short did not change significantly (Fig. 7A). Strikingly, phosphorylation of myosin II by TRPM7 was greatly reduced when the regulatory domain was deleted (Fig. 7B, Suppl. Fig. S1). The level of myosin II phosphorylation was only 5% of the levels achieved when using TRPM7-C-long in *in vitro* kinase reactions (Fig. 7C) despite TRPM7-C-short having similar catalytic activity in comparison to TRPM7-C-long (Fig. 7D). Removal of a more upstream region (aa 1158 to 1380) led to a kinase that underwent extensive electrophoretic mobility shift and could phosphorylate myosin II to similar degree as the TRPM7-C-long kinase (unpublished data). Thus, the autophosphorylation sites concentrated in the Ser/Thr-rich domain are responsible for the electrophoretic mobility shift and required for efficient phosphorylation of substrates but dispensable for catalytic activity.

### Despite a lack of sequence conservation, TRPM6 is also regulated by autophosphorylation of the Ser/Thr-rich domain

**Figure 8:**
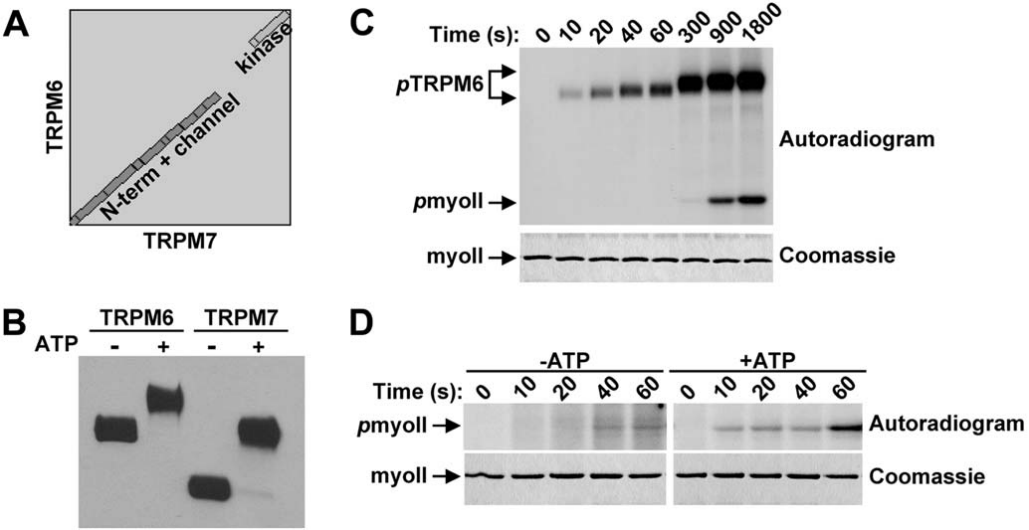
Regulation of TRPM6 by autophosphorylation. (A) TRPM6 and TRPM7 display no sequence homology in the region spanning between the TRP box and the kinase domain. Alignment of amino acid sequences of TRPM6 and TRPM7 using Blast-2-Blast from NCBI. The gap is a result of low sequence similarity between the two proteins in the indicated region. (B) Autophosphorylation of TRPM6 leads to a dramatic shift in electrophoretic mobility on SDS-PAGE gels. TRPM6-C-long and TRPM7-C-long were incubated in the absence or presence of MnATP, resolved by SDS-PAGE and subsequently detected using anti-HA antibodies. (C) TRPM6 autophosphorylation precedes myosin II phosphorylation. TRPM6-C-long was incubated with GST-myosin IIB in the presence of Mn[γ−^32^P]-ATP for the indicated times. The proteins were separated by SDS-PAGE, revealed by coommassie staining (bottom panel) and phosphorylated proteins were detected by autoradiography (top panel). (D) Accelerated kinetics of myosin II phosphorylation after pre-incubation of TRPM6 with ATP. TRPM6-C-long was incubated in the absence or presence of MnATP to generate non-phosphorylated and completely autophosphorylated TRPM6. To initiate the phosphorylation of myosin II, *in vitro* kinase buffer containing Mn[γ−^32^P]-ATP and GST-myosin IIB was added. The reaction was allowed to proceed at 30°C and stopped by adding Laemmli buffer after the indicated times. Proteins were resolved by SDS-PAGE, visualized by coomassie staining (bottom panel) and phosphorylated proteins were detected by autoradiography (top panel).

Alignment of the TRPM6 and TRPM7 sequences reveals that the protein is highly conserved throughout the molecule except in the region between the TRP and kinase domains of the C-terminal tail (Fig. 8A). Since this region regulates substrate recognition by TRPM7, we investigated whether it has a similar role in controlling TRPM6 kinase activity. Incubation of TRPM6-C-long with ATP led to a noticeable shift in electrophoretic mobility on SDS-PAGE gels (Fig. 8B). The kinetics of autophosphorylation of TRPM6-C-long also preceeded the phosphorylation of myosin II (Fig. 8C). Moreover, pre-incubation of TRPM6-C-long with ATP increased the rate of myosin II phosphorylation indicating that TRPM6 is activated by autophosphorylation (Fig. 8D). Despite the lack of sequence homology between TRPM6 and TRPM7, autophosphorylation also occurs in the region immediately upstream of the kinase (data not shown). Accordingly, this domain is serine and threonine-rich in both kinases. We conclude that although the regulatory domains of TRPM6 and TRPM7 show little similarity at the amino acid level, these sequences are essential for substrate phosphorylation by both kinases.

## Discussion

TRPM6 and TRPM7 play a key role in Mg^2+^-homeostasis [6]–[8]. In addition, TRPM7 has been implicated in other cellular processes including exocytosis, actomyosin contractility and cell adhesion [11]. The role of TRPM6 and TRPM7 kinase activity during these processes is largely unknown but experimental evidence has clearly demonstrated that it is dispensable for channel activation [18], [19]. Instead, the catalytic activity of TRPM6 and TRPM7 kinases is most likely required for the phosphorylation of downstream targets such as annexin I and myosin II [10], [31]. Our work now demonstrates a vital role for autophosphorylation of the Ser/Thr-rich domain in controlling the protein kinase activity of TRPM6 and TRPM7 towards their substrates. This investigation provides the first account of a mode of activation for the mammalian α-kinases TRPM6 and TRPM7 which will help refine current models and develop new ones as to how TRPM6 and TRPM7 function during health and disease.

How does autophosphorylation of the Ser/Thr-rich region affect the protein kinase activity of TRPM6 and TRPM7? Our data clearly demonstrate that in the absence of autophosphorylation or the Ser/Thr-rich domain, the phosphorylation of protein substrates by TRPM6 and TRPM7 is inefficient despite the kinase having full enzymatic activity. Thus, autophosphorylation of the Ser/Thr-rich region does not enhance enzymatic activity but provides access of the catalytic domain to the substrate. The simplest mechanistic explanation for this process is that substrate recognition occurs through a direct interaction between the hyperphosphorylated Ser/Thr-rich region and substrate. Unfortunately, the high salt conditions required to maintain myosin II filaments in a non-aggregated state *in vitro*, do not allow us to study direct interactions between hyperphosphorylated TRPM7 and myosin II. However, in support of this model, we have demonstrated that several residues in the Ser/Thr-rich domain are phosphorylated *in vivo* and that the association of TRPM7 with myosin II in mammalian cells requires kinase activity [10]. Alternatively, hyperphosphorylation of the Ser/Thr-rich region is predicted to cause dramatic changes in the 3D structure of TRPM6 and TRPM7, which may promote a conformation more apt for the phosphorylation of protein substrates. Current efforts are aimed at understanding the molecular mechanisms by which hyperphosphorylation of the Ser/Thr-rich domain elevates substrate phosphorylation.

Although TRPM6 and TRPM7 are highly homologous proteins (50% identity; 64% similarity), remarkably the Ser/Thr-rich domain is poorly conserved at the amino acid level. Yet TRPM6 also requires massive autophosphorylation of the Ser/Thr-rich region for efficient substrate phosphorylation. From these results, we conclude that the build-up of negative charge in response to hyperphosphorylation is the defining property rather than the primary structure of the protein. In an attempt to further examine this notion, we varied the salt concentration in the kinase reactions. It is predicted that if the negative charge is an important characteristic of the hyperphosphorylated kinase then counteracting this charge by adding sodium ions should interfere with protein phosphorylation. Indeed, we noticed that the addition of salt inhibited the ability of TRPM7-C-long to phosphorylate myosin II but it had no effect on autophosphorylation (Suppl. Fig. S2A, B). The differences in the degree of TRPM7-mediated phosphorylation of itself and myosin II in presence of salt is not due to different modes of catalysis (intra- vs. intermolecular) since intermolecular autophosphorylation of TRPM7 appears to be independent of salt or the presence of the regulatory domain (Suppl. Fig. S2C). These data further strengthen the notion that the role of hyperphosphorylation of the Ser/Thr-rich region is to allow access of the catalytic domain to the substrate and moreover, that this process requires the build-up of negative charge. Notably, the region of the coiled-coil domain of myosin II that is phosphorylated by TRPM7 is positively charged. Thus, we speculate that electrostatic interactions between the hyperphosphorylated Ser/Thr-rich domain and myosin II are established to enhance substrate phosphorylation or provide substrate specificity *in vivo*. Although we cannot exclude other effects of salt within the kinase reactions such as effects on substrate rather than kinase, this salt dependency should be further explored to assess the role of massive autophosphorylation in regulating the kinase activity of TRPM6 and TRPM7 towards their substrates.

One of the most surprising results of this investigation is the unprecedented number of autophosphorylation sites in TRPM6 and TRPM7. By LC-MS/MS, we identified 46 residues phosphorylated in wild-type (WT) but not in a catalytically inactive mutant of TRPM7 demonstrating that phosphorylation the result of autophosphorylation. Moreover, hyperphosphorylation of TRPM7 appears to be a physiologically relevant process since 20 sites were phosphorylated *in vivo.* Our phosphomapping experiments are consistent with the level of phosphate incorporation into the kinase where 32±4 mol of phosphate is incorporated per mol of TRPM7. Moreover, we identified previously reported sites of TRPM7 autophosphorylation including Thr1482, Ser1512 and Ser1568 [18], [32]. The authenticity of the phosphorylation sites is further demonstrated by the ratio of phosphorylated serine and threonine residues and their distribution within TRPM7. Phosphoamino acid analysis of TRPM7 determined that phosphate is incorporated 70% into serines and 30% into threonines [28], [32]. Accordingly, we identify 31 serine and 15 threonine residues that are phosphorylated by TRPM7. Moreover, these residues (37 out of 46) are predominantly located in the Ser/Thr-rich domain, which is directly adjacent to the catalytic domain. Deletion of this region leads to a corresponding 4-fold decrease in phosphate incorporation into TRPM7. Others have also found this region of TRPM6 and TRPM7 to be strongly autophosphorylated [18], [28]. In conclusion, our data convincingly show the presence and identification of multiple TRPM6/TRPM7 autophosphorylation sites.

A major challenge is to identify residues essential for TRPM6 and TRPM7 kinase activity. By deletion mapping, we have determined that the Ser/Thr-rich region between residues 1380 and 1548, which contains the greatest density of autophosphorylation sites, is required for the electrophoretic mobility shift and efficient phosphorylation of substrates. However, we have yet to identify any priming event that would modulate the extent of TRPM7 autophosphorylation, which is consistent with a model where a build-up of negative charge is required for substrate phosphorylation. Mutation of different residues to alanine (Ser1512, Ser1568, Thr1686, Ser1694, Ser1780) either individually or in combination did not suffice to affect TRPM7 autophosphorylation or activity towards myosin II (Suppl. Fig. S3). Previously, Matsushita and colleagues reported that Ser1512 and Ser1568 are the major sites of TRPM7 autophosphorylation [18]. Mutation of these residues to alanine led to a 90% decrease in phosphate incorporation during *in vitro* kinase assays. This discrepancy with our study may reflect differences in expression systems. We have opted to purify TRPM7 directly from mammalian cells whereas Matsushita *et al.* [18] produced TRPM7 in bacteria. A major problem with the purification of recombinant proteins from prokaryotic cells is the reproducibility of the specific activity of the enzyme. Unfortunately, Matsushita and co-workers [18] did not provide evidence that catalytic activity of the TRPM7 S1512A/S1568A double mutant was intact by measuring the phosphorylation of a peptide substrate such as MBP, which was previously shown to be independent of autophosphorylation [29], nor did they confirm that mutation of Ser1512 and Ser1568 was sufficient to abolish the incorporation of ^32^P into TRPM7 in mammalian cells. In contrast, we have found many autophosphorylation sites of TRPM7 basally phosphorylated in mammalian cells and in combination with our *in vitro* analysis of TRPM7 mutants, it seems unlikely that the TRPM7 S1512A/S1568A mutant would have significantly reduced incorporation of phosphate in living cells. Although our study raises doubts about the contribution of Ser1512 and Ser1568 to the capacity of TRPM7 to undergo autophosphorylation in mammalian cells, it is plausible that an unknown factor in mammalian cells may predispose TRPM7 to undergo massive autophosphorylation despite the mutation of Ser1512 and Ser1568 to alanine. This factor could be an associated protein or phosphorylation by upstream protein kinases. Indeed, we have found 3 phosphorylation sites in a catalytically inactive mutant of TRPM7 demonstrating the presence of protein kinases capable of phosphorylating TRPM7. These sites are unlikely to be phosphorylated in bacterially-produced TRPM7 and therefore, may regulate the extent of TRPM7 autophosphorylation. Clearly, further studies are required to address the discrepancies between our study and that of Matsushita and colleagues [18].

The molecular mechanism regulating the protein kinase activity of TRPM6 and TRPM7 shows similarities but also important differences with that of other α-kinases. The *Dictyostelium* myosin heavy chain kinases (MHCK) A, B and C are all activated by autophosphorylation [33]–[35]. The level of autophosphorylation displays a wide dynamic range with stoichiometry of phosphorylation ranging from 6–20 mol Pi/ mol kinase [33], [34], [36]. However, complete autophosphorylation does not appear to be required for activation of MHC kinases since autophosphorylation of 3 sites (out of 10) is sufficient to activate MHCKA [33] and myosin II phosphorylation proceeds in parallel to MHCKC autophosphorylation [34]. In contrast, TRPM6 and TRPM7 only phosphorylate myosin II under conditions allowing complete autophosphorylation of the kinases. Interestingly, eukaryotic elongation factor-2 (eEF-2) kinase and MHCK, like TRPM7, require the presence of additional regulatory domains for efficient substrate phosphorylation. In contrast to TRPM6 and TRPM7 however, the regulatory domains in eEF-2 kinase and MHCK are not subject to autophosphorylation [36]–[38]. Similar to deletion of the Ser/Thr-rich domain of TRPM6 and TRPM7, removal of the regulatory domains in eEF-2 kinase and MHCK decreases the rate of substrate phosphorylation without affecting the kinetics of phosphorylation of a synthetic peptide [36]–[39]. We therefore speculate that the Ser/Thr-rich domain has a similar function to the regulatory domains found in other α-kinases which play a key role in substrate recognition and that autophosphorylation of this region provides an additional level of regulation of TRPM6 and TRPM7 protein kinase activity.

In conclusion, we have identified a novel mechanism regulating the activity of the mammalian α-kinases TRPM6 and TRPM7. Based on the experimental evidence, we propose that massive autophosphorylation of the Ser/Thr-rich domain in TRPM6 and TRPM7 provides an electrostatic interface for the recruitment of substrates which greatly enhances their phosphorylation. Therefore, the protein kinase activity of TRPM6 and TRPM7 may impinge on their cellular functions by controlling the recruitment of substrates *in vivo* through hyperphosphorylation of the Ser/Thr-rich domain. Future experiments should be aimed at revealing the structural changes that occur in TRPM6 and TRPM7 after autophosphorylation and understanding how these contribute to phosphorylation of substrates.

## Materials and Methods

### Constructs

To generate the different expression vectors encoding human TRPM6 and TRPM7 kinase variants (Fig. 1), cDNA was amplified by PCR using primers containing an HA-tag at the 5′-end and inserted into pcDNA3 as a *BamHI-NotI* fragment. Point mutations were introduced into TRPM7 by site-directed mutagenesis using the Quikchange mutagenesis kit (Stratagene). Human myosin IIA (aa 1795–1960) and IIB (aa 1802–1977) heavy chain tails were amplified by PCR and inserted into the *BamHI-EcoRI* sites in frame with GST in the pGEX-1N vector. All constructs were verified by DNA sequencing.

### Cell culture

HEK293 cells were cultured in DMEM medium with 10% FCS. Cells were transfected using Lipofectamine 2000 (Invitrogen) according to the manufacturer's recommendations.

### Purification of GST-myosin II and protein kinases

GST-myosin IIA and GST-myosin IIB were expressed in *E. Coli* and purified by affinity chromatography on a glutathione-sepharose column using standard methods. TRPM6 and TRPM7 kinases (Fig 1) were purified from mammalian cells by immunoprecipitation. Cells were lysed in RIPA buffer and the kinases were immunoprecipitated using anti-HA antibodies (12CA5) coupled to protein G-sepharose.

### In vitro kinase assays

Immunecomplexes containing 20 ng of kinase were washed and resuspended in IVK buffer (50 mM HEPES pH 7.0, 4 mM MnCl_2_, 2 mM DTT) with or without substrate. For protein substrates, 2 µg of GST-myosin IIA, GST-myosin IIB or myelin basic protein (MBP) were added to the reaction whereas the peptide substrate MH1 (RKKFGESEKTKTKEFL) was present at a concentration of 100 µM. The kinase reactions were initiated by adding MnATP and were incubated at 30°C for the indicated times. Kinase reactions containing recombinant proteins as substrates were stopped by the addition of Laemmli buffer containing 40 mM EDTA and subjected to SDS-PAGE. Proteins were detected by immunoblotting using anti-HA (3F10, Roche) or anti-phosphothreonine antibodies (Cell Signalling Technology) or by autoradiography. Quantification was performed by phosphorimager analysis. Peptide substrates were spotted on p81 cellulose paper and quantified by scintillation counting.

### Mass Spectrometry

Phosphoproteins were separated by SDS-PAGE, stained with colloidal coomassie blue and digested in-gel with trypsin using standard protocols. Peptide identification experiments were performed using a nano HPLC Agilent 1100 nano flow system connected online to a 7-Tesla linear ion trap Ion Cyclotron Resonance Fourier Transform (LTQ-FT) mass spectrometer (Thermo Fisher, Bremen, Germany). Verification and site mapping of phosphorylated peptides was perfomed according to the procedure of Olsen and coworkers [30]. Phosphopeptides were identified with a 99%-significance threshold when the sum of the Mascot and PTM score was higher than 28 [30]. The delta PTM score, the difference between the highest and second highest PTM score, was set to be larger than 6.5 to exclusively report peptides with a mapped phosphorylation site. Moreover, only phosphopeptides occurring more than once were included in the results.

## Supporting Information

Table S1 TRPM7 phosphopeptides detected in WT and KD TRPM7-C-long by LC-MS/MS.
(0.20 MB XLS)

Figure S1 Autophosphorylation and substrate phosphorylation by TRPM7-C-short is not due to a contaminating protein kinase. WT and KD TRPM7-C-short were incubated with GST-myosin IIB in presence of Mn[g-32P]-ATP. Proteins were separated by SDS-PAGE and detected by coomassie staining (middle panel). Phosphorylated proteins were revealed by autoradiography (top panel). Equal amounts of WT and KD TRPM7-C-short was verified by Western blotting (bottom panel).
(0.33 MB TIF)

Figure S2 High ionic strength inhibits substrate phosphorylation by TRPM7 without affecting catalytic activity. (A) Effect of salt on TRPM7 and myosin II phosphorylation. TRPM7-C-long was incubated with GST-myosin IIB and Mn[g-32P]-ATP in the absence or presence of 250 mM NaCl for 30 min at 30°C. The proteins were separated by SDS-PAGE, stained with coomassie brilliant blue (bottom panel) and phosphorylated proteins were detected by autoradiography (top panel). (B) Quantification of TRPM7 and myosin II phosphorylation levels in absence and presence of salt. 32P incorporation was measured by phosphorimaging analysis. The phosphorylation levels measured for the sample containing no salt was set to 1 for each protein and values obtained in presence of salt are reported relative to this value. (C) Intermolecular autophosphorylation of TRPM7 is independent of salt. Lysates of mammalian cells expressing WT TRPM7-C-short were added to those of cells expressing KD TRPM7-C-long. The proteins were co-purified by immunoprecipitation using anti-HA antibodies. The beads were incubated with Mn[g-32P]-ATP for 30 min at 30°C in presence of 0 and 250 mM NaCl. Proteins were separated by SDS-PAGE and phosphorylated proteins were detected by autoradiography.
(0.46 MB TIF)

Figure S3 Mutation of Ser1512, Ser1568, Thr1686, Ser1694 or Ser1780 to alanine has no effect on TRPM7 autophosphorylation or substrate phosphorylation. TRPM7-C-long constructs containing the point mutations S1512A, S1568A, T1686A, S1694A or S1780A and the double mutants S1512A/S1568A and T1686A/S1780A were immunoprecipitated from mammalian cells and subjected to in vitro kinase assays. (A) Electrophoretic mobility shift. After incubation in absence or presence of ATP, proteins were subjected to immunoblotting using anti-HA antibodies. (B) Phosphorylation of myosin II is unaffected by the different point mutations. TRPM7-C-long was incubated with GST-myosin IIB in presence of Mn[g-32P]-ATP. Proteins were separated by SDS-PAGE and detected by coomassie staining (middle panel). Phosphorylated proteins were revealed by autoradiography (top panel). Equal amounts of WT and mutant TRPM7 kinase was verified by Western blotting (bottom panel).
(1.10 MB TIF)
