# Supplementary figures and images for: Massive Autophosphorylation of the Ser/Thr-Rich Domain Controls Protein Kinase Activity of TRPM6 and TRPM7
Source: PLoS One. 2008 Mar 26;3(3):e1876. doi: 10.1371/journal.pone.0001876 (PMC2267223; doi:10.1371/journal.pone.0001876)

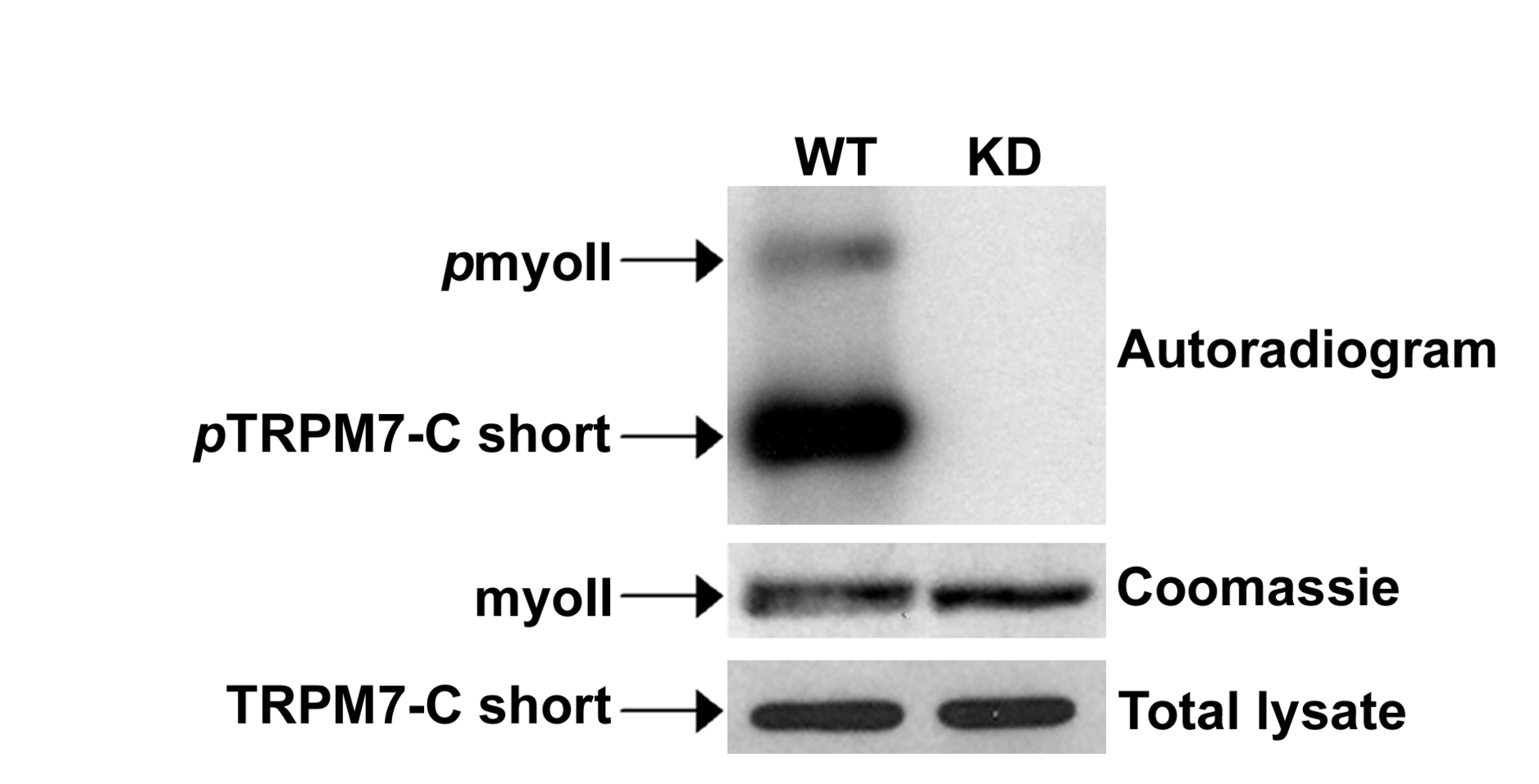

Supplement: Figure S1 — Autophosphorylation and substrate phosphorylation by TRPM7-C-short is not due to a contaminating protein kinase. WT and KD TRPM7-C-short were incubated with GST-myosin IIB in presence of Mn[g-32P]-ATP. Proteins were separated by SDS-PAGE and detected by coomassie staining (middle panel). Phosphorylated proteins were revealed by autoradiography (top panel). Equal amounts of WT and KD TRPM7-C-short was verified by Western blotting (bottom panel). (0.33 MB TIF) [file pone.0001876.s002.tif]

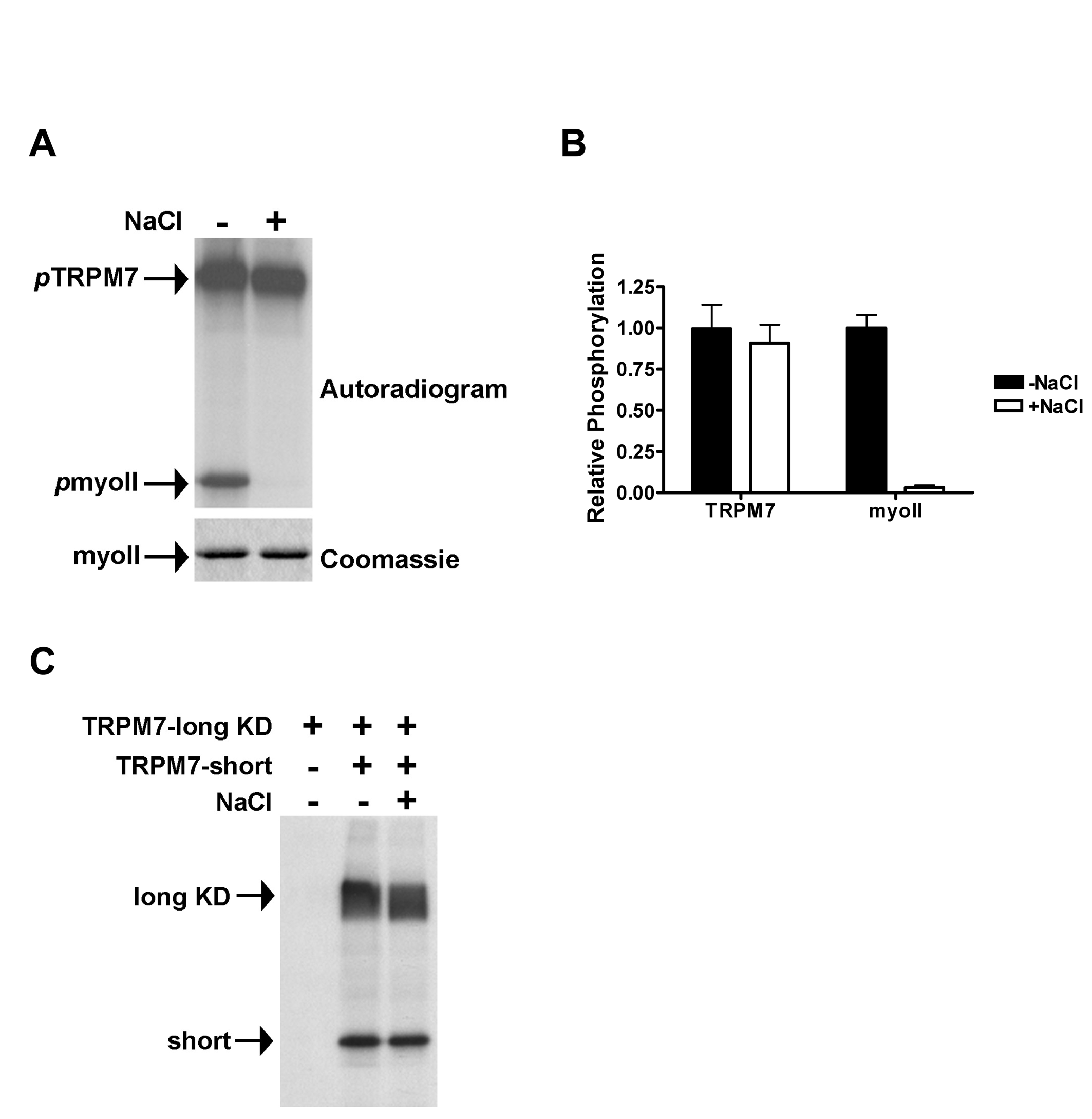

Supplement: Figure S2 — High ionic strength inhibits substrate phosphorylation by TRPM7 without affecting catalytic activity. (A) Effect of salt on TRPM7 and myosin II phosphorylation. TRPM7-C-long was incubated with GST-myosin IIB and Mn[g-32P]-ATP in the absence or presence of 250 mM NaCl for 30 min at 30°C. The proteins were separated by SDS-PAGE, stained with coomassie brilliant blue (bottom panel) and phosphorylated proteins were detected by autoradiography (top panel). (B) Quantification of TRPM7 and myosin II phosphorylation levels in absence and presence of salt. 32P incorporation was measured by phosphorimaging analysis. The phosphorylation levels measured for the sample containing no salt was set to 1 for each protein and values obtained in presence of salt are reported relative to this value. (C) Intermolecular autophosphorylation of TRPM7 is independent of salt. Lysates of mammalian cells expressing WT TRPM7-C-short were added to those of cells expressing KD TRPM7-C-long. The proteins were co-purified by immunoprecipitation using anti-HA antibodies. The beads were incubated with Mn[g-32P]-ATP for 30 min at 30°C in presence of 0 and 250 mM NaCl. Proteins were separated by SDS-PAGE and phosphorylated proteins were detected by autoradiography. (0.46 MB TIF) [file pone.0001876.s003.tif]

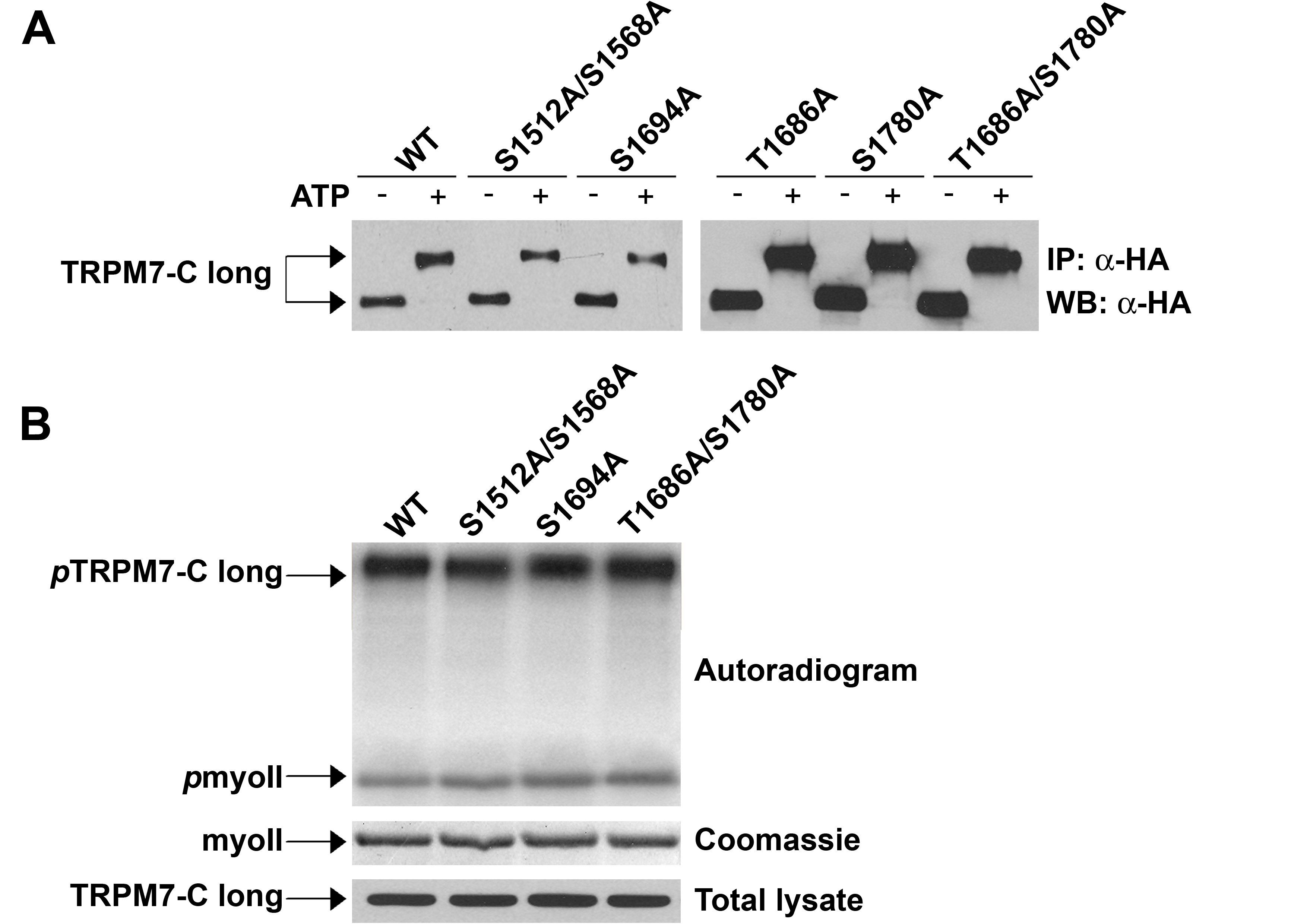

Supplement: Figure S3 — Mutation of Ser1512, Ser1568, Thr1686, Ser1694 or Ser1780 to alanine has no effect on TRPM7 autophosphorylation or substrate phosphorylation. TRPM7-C-long constructs containing the point mutations S1512A, S1568A, T1686A, S1694A or S1780A and the double mutants S1512A/S1568A and T1686A/S1780A were immunoprecipitated from mammalian cells and subjected to in vitro kinase assays. (A) Electrophoretic mobility shift. After incubation in absence or presence of ATP, proteins were subjected to immunoblotting using anti-HA antibodies. (B) Phosphorylation of myosin II is unaffected by the different point mutations. TRPM7-C-long was incubated with GST-myosin IIB in presence of Mn[g-32P]-ATP. Proteins were separated by SDS-PAGE and detected by coomassie staining (middle panel). Phosphorylated proteins were revealed by autoradiography (top panel). Equal amounts of WT and mutant TRPM7 kinase was verified by Western blotting (bottom panel). (1.10 MB TIF) [file pone.0001876.s004.tif]
